# Metformin Induces Apoptosis through AMPK-Dependent Inhibition of UPR Signaling in ALL Lymphoblasts

**DOI:** 10.1371/journal.pone.0074420

**Published:** 2013-08-23

**Authors:** Gilles M. Leclerc, Guy J. Leclerc, Jeffim N. Kuznetsov, Joanna DeSalvo, Julio C. Barredo

**Affiliations:** 1 Department of Pediatrics Hematology and Oncology, University of Miami Miller School of Medicine, Miami, Florida, United States of America; 2 Department of Ophthalmology, University of Miami Miller School of Medicine, Miami, Florida, United States of America; 3 Department of Biochemistry and Molecular Biology, University of Miami Miller School of Medicine, Miami, Florida, United States of America; 4 Sylvester Comprehensive Cancer Center, University of Miami Miller School of Medicine, Miami, Florida, United States of America; The Ohio State University, United States of America

## Abstract

The outcome of patients with resistant phenotypes of acute lymphoblastic leukemia (ALL) or those who relapse remains poor. We investigated the mechanism of cell death induced by metformin in Bp- and T-ALL cell models and primary cells, and show that metformin effectively induces apoptosis in ALL cells. Metformin activated AMPK, down-regulated the unfolded protein response (UPR) demonstrated by significant decrease in the main UPR regulator GRP78, and led to UPR-mediated cell death via up-regulation of the ER stress/UPR cell death mediators IRE1α and CHOP. Using shRNA, we demonstrate that metformin-induced apoptosis is AMPK-dependent since AMPK knock-down rescued ALL cells, which correlated with down-regulation of IRE1α and CHOP and restoration of the UPR/GRP78 function. Additionally rapamycin, a known inhibitor of mTOR-dependent protein synthesis, rescued cells from metformin-induced apoptosis and down-regulated CHOP expression. Finally, metformin induced PIM-2 kinase activity and co-treatment of ALL cells with a PIM-1/2 kinase inhibitor plus metformin synergistically increased cell death, suggesting a buffering role for PIM-2 in metformin’s cytotoxicity. Similar synergism was seen with agents targeting Akt in combination with metformin, supporting our original postulate that AMPK and Akt exert opposite regulatory roles on UPR activity in ALL. Taken together, our data indicate that metformin induces ALL cell death by triggering ER and proteotoxic stress and simultaneously down-regulating the physiologic UPR response responsible for effectively buffering proteotoxic stress. Our findings provide evidence for a role of metformin in ALL therapy and support strategies targeting synthetic lethal interactions with Akt and PIM kinases as suitable for future consideration for clinical translation in ALL.

## Introduction

Acute Lymphoblastic Leukemia (ALL), the most common malignancy in children and adolescents, remains the number one cause of cancer-related death for patients under the age of twenty [1]. Despite significant overall improvements in cure rates, outcome remains poor for patients with resistant phenotypes or after relapse, and long-term treatment-related morbidity can be significant for survivors of childhood ALL [2]. Consequently, novel and less toxic treatment strategies are needed to improve cure rates and decrease long-term sequelae for these patients. We identified the AMP activated protein kinase (AMPK), a regulator of energy homeostasis in eukaryotic cells [3], as a target for ALL therapy due to its effects on cell growth and cell cycle regulation, as well as its crosstalk with critical metabolic and oncogenic pathways [4]. AMPK is a heterotrimeric complex composed of a catalytic α subunit and two regulatory subunits (β and γ) [5]. AMPK is activated by metabolic stressors that deplete ATP and increase AMP, and by upstream kinases [6] that induce its phosphorylation at Thr172 [7]. Activated AMPK down-regulates processes that consume ATP (cell growth and protein synthesis) and activate pathways responsible for the generation of energy such as glycolysis and fatty acid oxidation [8].

The biguanide drug metformin (N,N-Dimethylimidodicarbonimidic diamide), currently used for treatment of diabetes [9], is known to activate AMPK. Metformin has been shown to induce metabolic stress by various mechanisms including inhibition of AMP deaminase [10] and the mitochondrial respiration chain complex 1 [11], both of which lower the ATP: AMP ratio leading to AMPK activation. Although metformin is linked to lower incidence of cancer and induction of cell death in various solid tumor types [12–14], its mechanism of cell death has not been fully investigated in leukemia. We and others have reported that AMPK can act as a physiological suppressor of the unfolded protein response (UPR) following exposure to AMPK activators such as AICAR [15,16], metformin [17,18], or the glycolytic inhibitor 2-deoxy-D-glucose (2-DG) [19]. This homeostatic mechanism is triggered in response to the accumulation of unfolded/misfolded proteins in the ER lumen [20]. The UPR is mediated via three ER transmembrane receptors: protein kinase dsRNA-like ER kinase (PERK), activating transcription factor 6 (ATF6), and inositol-requiring enzyme 1 (IRE1) [21]. These receptors are activated upon dissociation from the main ER chaperone protein GRP78 to fully engage the UPR function, which encompasses blocking of protein synthesis (via phosphorylation of eIF2α), activation of proteasomal protein degradation, and transcriptional induction of ER chaperone genes (GRP78 and GRP94) as well as the pro-apoptotic transcription factor CHOP (CCAAT/enhancer binding protein homologous) [22]. In addition, GRP78 functions to suppress pro-apoptotic pathways of the UPR via activation of Akt and Erk signaling [23,24]. During sustained ER stress, the pro-apoptotic arm of the UPR activates IRE1α, CHOP, caspases, the apoptotic signaling-kinase-1 (ASK1) and its downstream target c-Jun NH_2_-terminal kinase (JNK) [25,26]. Therefore, both a functional anti-apoptotic and pro-apoptotic arm are ascribed to the UPR [27].

In mammalian cells, protein translation is mainly regulated by the mammalian target of rapamycin (mTOR), which phosphorylates among others the two key protein translation regulators p70S6K and 4-EBP1 [28]. Phosphorylation of the latter promotes its dissociation from the translational regulator eukaryotic initiation factor 4E allowing cap-dependent translation [29]. Recently, PIM kinases have been shown to regulate cell growth, energy metabolism, and programmed cell death through interactions with 4-EBP1 [30], AMPK [31], and BAD [32], respectively. The PIM kinase family consists of three oncogene-encoded serine/threonine kinases (PIM-1/2/3) [33] that are frequently overexpressed in human hematological and solid malignancies [34]. In most hematopoietic malignancies, PIM expression has been correlated with poor prognosis, underscoring the clinical significance of PIM kinases in cancer biology [35].

In this study, we used pharmacological and genetic approaches to investigate the mechanism of cell death induced by metformin in ALL. Our data indicate that metformin activates AMPK and induces apoptosis by triggering ER stress and preventing ALL cells from engaging the UPR in order to effectively buffer an increased proteotoxic load due to the accumulation of unfolded proteins in the ER lumen. Thus, metformin triggers the UPR-mediated apoptotic pathway via an AMPK-dependent mechanism.

## Material and Methods

### Ethics statement

This study was conducted according to the principles expressed in the Declaration of Helsinki. The study was approved by the University of Miami Human Subject Research Office (HSRO) (IRB protocol # 20110555). Primary ALL samples were obtained from patients with ALL at the University of Miami following Institutional Review Board (IRB) approved written informed consent by the University of Miami Human Subject Research Office (HSRO).

### Cell culture and reagents

The ALL cell lines CCRF-CEM (T-ALL), Jurkat (T-ALL), and REH (B precursor ALL, Bp-ALL, t(12;21)/TEL-AML1) were obtained from the American Type Culture Collection (ATCC; http://www.atcc.org, Manassas, VA). NALM6 (Bp-ALL) cell line was obtained from the Leibniz Institute Deutsche Sammlung von Mikroorganismen und Zellkulturen GmbH (DSMZ, https://www.dsmz.de, Braunschweig, Germany). These cell lines were maintained in RPMI-1640 medium (Cellgro, Manassas, VA) supplemented with 10% heat inactivated fetal bovine serum (Sigma-Aldrich, St. Louis, MO) and antibiotics (penicillin, 100 I.U. /ml; streptomycin, 100 µg/ml) (Cellgro) at 37°C under an atmosphere of 5% CO_2_. Primary ALL cells were co-cultured using a human bone marrow stromal cell feeder layer as described previously [19]. Metformin and tunicamycin were purchased from Sigma-Aldrich, rapamycin from LC Laboratories (Woburn, MA), and perifosine from Selleck Chemicals (Houston, TX). The Akt Inhibitor X and the PIM-1/2 kinase inhibitor V were obtained from Calbiochem (Gibbstown, NJ).

### Cell viability and apoptosis assays

Logarithmic cells were exposed to the indicated drugs for up to 72 h. At specified time points, cell proliferation and cell death were determined using the Vi-Cell XR system (trypan blue exclusion) (Beckman Coulter, Brea, CA) and expressed as a percentage relative to control values or a percentage of dead cells in the population (mean ±SEM, n=3), respectively. Apoptosis was determined by flow cytometric analysis of Annexin V-FITC/propidium iodide (PI) staining as described previously [36]. The Annexin V and PI data were combined and expressed as a percentage of apoptosis (mean ± SEM, n=3). Synergism was determined by calculating the combination index (CI) using the following equation: CI = [(D1-combination/D1-single) + (D2-combination/D2-single)] as described by Chou [37]. Statistical significance in cell growth or apoptosis data following drug treatments was assessed by one-way ANOVA followed by the Newman-Keuls multiple comparison tests or by unpaired Student’s *t* test using GraphPad PRISM (San Diego, CA).

### Construction of stable shRNA expressing cell lines

To down-regulate the expression of AMPK1α, we used a pool of lentiviral particles containing three target-specific constructs that encode specific shRNAs against AMPKα1 (shAMPK) (sc-296730V; Santa Cruz Biotechnology, Santa Cruz, CA). Control lentiviral shRNAs (shCTRL, sc-108080) were obtained from Santa Cruz. Stable CCRF-CEM cells expressing shAMPK and shCTRL were generated by transduction using lentiviral stock as described elsewhere [19].

### Protein extracts and Western immunoblots

Cells were sonicated in 1X RIPA (Thermo Scientific, Rockford, IL) containing Halt™ protease and phosphatase inhibitor cocktails (Thermo Scientific). Western blots were carried out as described elsewhere [15]. The primary antibodies and the secondary HRP-conjugated antibody were obtained from Cell Signaling (Beverly, MA).

## Results

### Metformin induces growth inhibition and cell death by altering AMPK, Akt/mTOR and UPR signaling pathways in ALL cell models and primary cells

**Figure 1:**
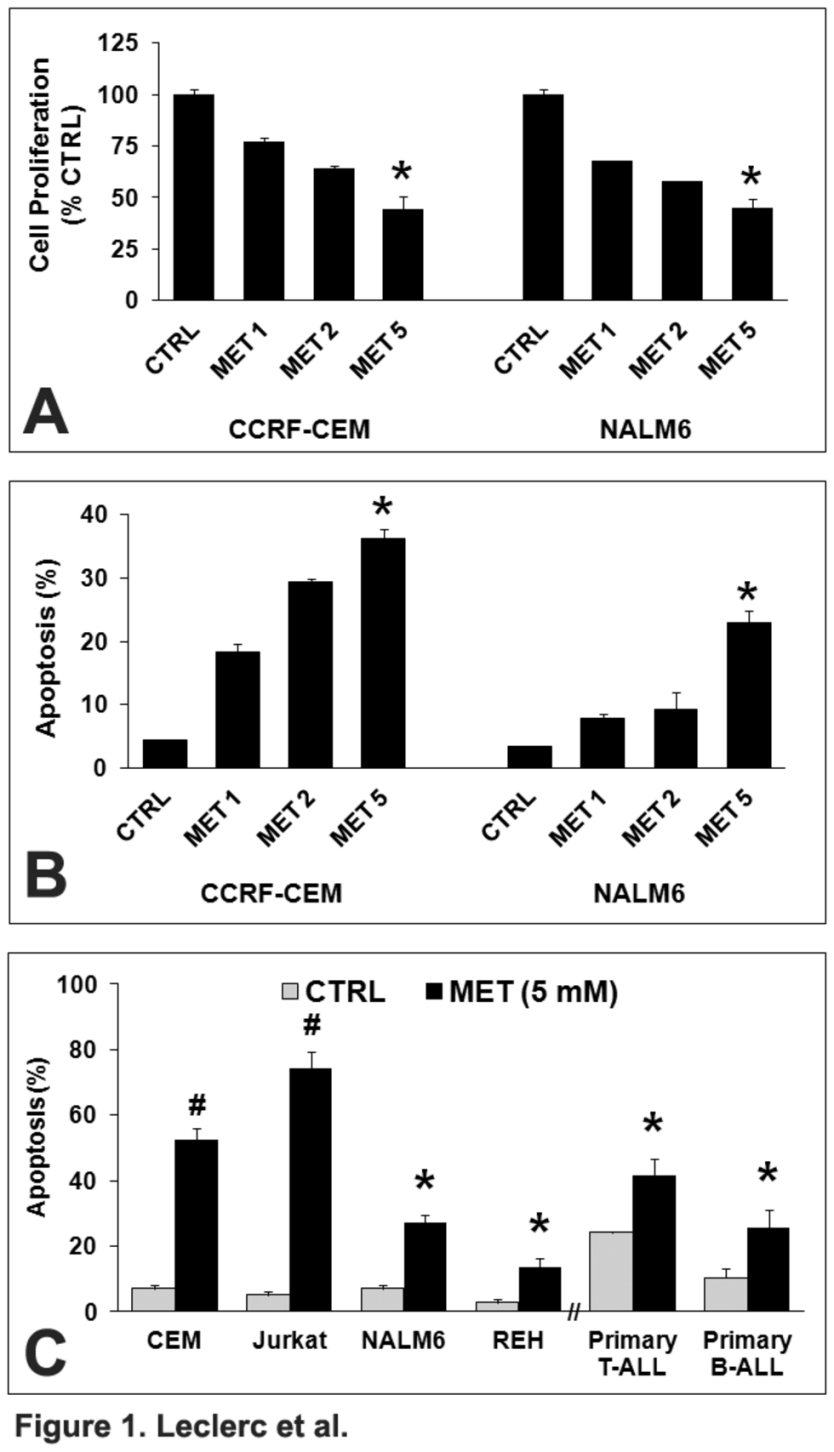
Metformin induces cell growth arrest and apoptosis in ALL cell lines. Growth inhibition (**A**) and apoptosis (**B**) in CCRF-CEM and NALM6 cells treated with metformin (MET, 1, 2, and 5 mM) for 48 h. Apoptosis (**C**) in T-ALL (CCRF-CEM, Jurkat, and primary T-ALL) and Bp-ALL (NALM6, REH, and primary Bp-ALL) cells treated with metformin (MET, 5 mM) for 48 h. Growth inhibition was expressed relative to control values (mean ±SEM, n = 3). * and # denote *p* < 0.001 and *p* < 0.0001 for metformin *vs*. CTRL, respectively.

To evaluate the clinical relevance of metformin in ALL, we determined its effects on ALL cell growth and death. The ALL cell models CCRF-CEM (T-ALL) and NALM6 (Bp-ALL) were treated with metformin and effects on cell proliferation and apoptosis determined at 48 h. As shown in Figure 1, metformin (1-5 mM) induced cell growth arrest (Figure 1A) and apoptosis (Figure 1B) in both CCRF-CEM and NALM6 cell lines. Similar data were obtained with other representative T-ALL (Jurkat) and Bp-ALL (REH) models and primary T- and Bp-ALL cells from children with ALL (Figure 1C), indicating that metformin induces cells death in both T- and Bp-ALL primary and cell line models.

**Figure 2:**
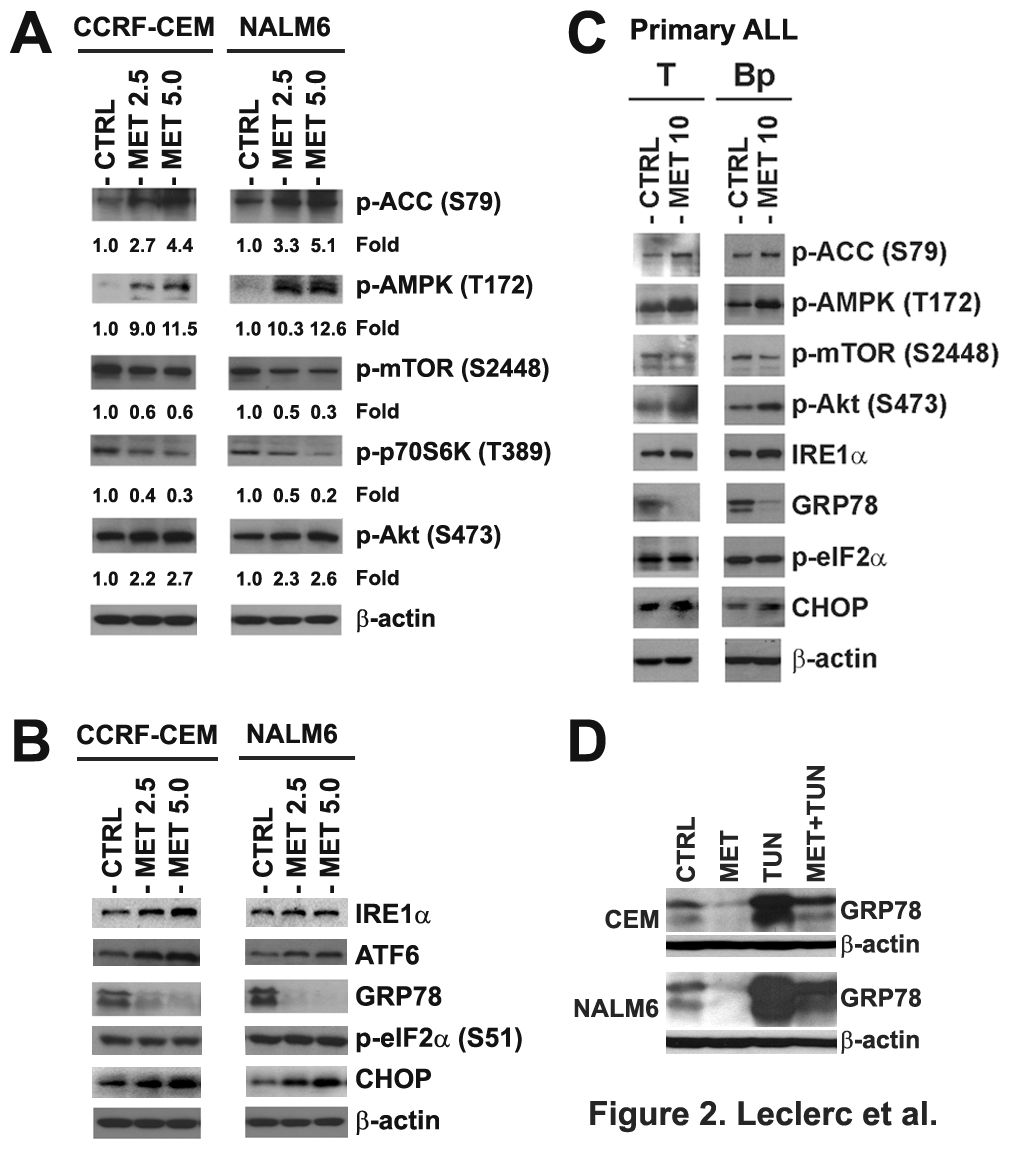
Metformin activates AMPK, Akt, and UPR signaling pathway proteins in ALL primary and cell line models. **A**) Western blot analysis of proteins associated with AMPK, and Akt/mTOR signaling pathways in CCRF-CEM and NALM6 cells treated with metformin (MET, 2.5 and 5.0 mM) for 48 h. The density value of each band was normalized to β-actin level and expressed relative to control (shown as fold induction). **B**) Immunoblotting of UPR signaling factors in CCRF-CEM and NALM6 cells treated with metformin (MET, 2.5 and 5.0 mM) for 48 h. **C**) Western blots of AMPK, Akt/mTOR and UPR signaling proteins in representative sample of primary T- and Bp-ALL cells treated with metformin (MET, 10 mM) for 24 h. **D**) Western blot analysis of GRP78 expression in CCRF-CEM and NALM6 cells treated with metformin (MET, 10mM) and tunicamycin (TUN, 2.5 µg/ml for NALM6; 5.0 µg/ml for CCRF-CEM), either alone or in combination for 48 h.

To understand the mechanism of cell death induced by metformin in T- and Bp-ALL cells, we examined the expression of critical signaling proteins associated with energy metabolism and cell proliferation pathways in CCRF-CEM and NALM6 cell lines treated with metformin. We found that metformin induced phosphorylation/activation of p-AMPK (T172), its downstream target p-ACC (S79), and phosphorylation/activation of Akt at Ser473 (Figure 2A). The activation of AMPK resulted in down-regulation of p-mTOR (S2448) and its target p-p70S6K (T389). These changes are consistent with those we previously reported with other AMPK activators [36].

Since we have previously showed that AMPK suppresses the UPR in ALL [19], and others had reported that metformin can inhibit the UPR in normal renal tubular epithelial cells and certain carcinomas [38,39], we determined the effects of metformin on the expression of UPR markers in cell lines and primary ALL cells. Western blot analysis of CCRF-CEM and NALM6 cells treated with metformin revealed a significant decrease in GRP78 expression (Figure 2B), indicating that metformin alters the regulation of the UPR. In addition, we detected increased expression of IRE1α, ATF6, and of the known ER stress/UPR-mediated cell death marker CHOP [40] in ALL cells treated with metformin (Figure 2B). Our results indicate that metformin induces ER stress/UPR mediated cell death and suggests the mechanism of apoptotic death may be triggered by the inability of ALL cells to adequately respond to ER and/or proteotoxic stress through the UPR. Similar signaling alterations were observed in metformin-treated representative primary T- and Bp-ALL cells, highlighting the clinical relevance of this putative mechanism of action (Figure 2C). To examine if the effect of metformin on GRP78 down-regulation represented a direct effect and was not simply a correlative finding, we co-treated CCRF-CEM and NALM6 cells with metformin and tunicamycin, a known ER stress inducer [41]. Tunicamycin activates GRP78 via inhibition of N-linked glycosylation which triggers ER stress/UPR due to the accumulation of misfolded proteins in the ER. As expected, tunicamycin alone strongly induced GRP78 expression in ALL cells (Figure 2D), whereas we found that metformin alone significantly reduced the level of GRP78. More important, when both drugs were used in combination, GRP78 expression was significantly decreased compared to cells treated with tunicamycin alone (Figure 2D), confirming a cause and effect relationship between metformin and down-regulation of GRP78 expression and UPR function in ALL cells.

### Down-regulation of AMPK rescues ALL cells from metformin-induced ER stress/UPR-mediated cell death by de-repressing the UPR and interrupting protein synthesis

**Figure 3:**
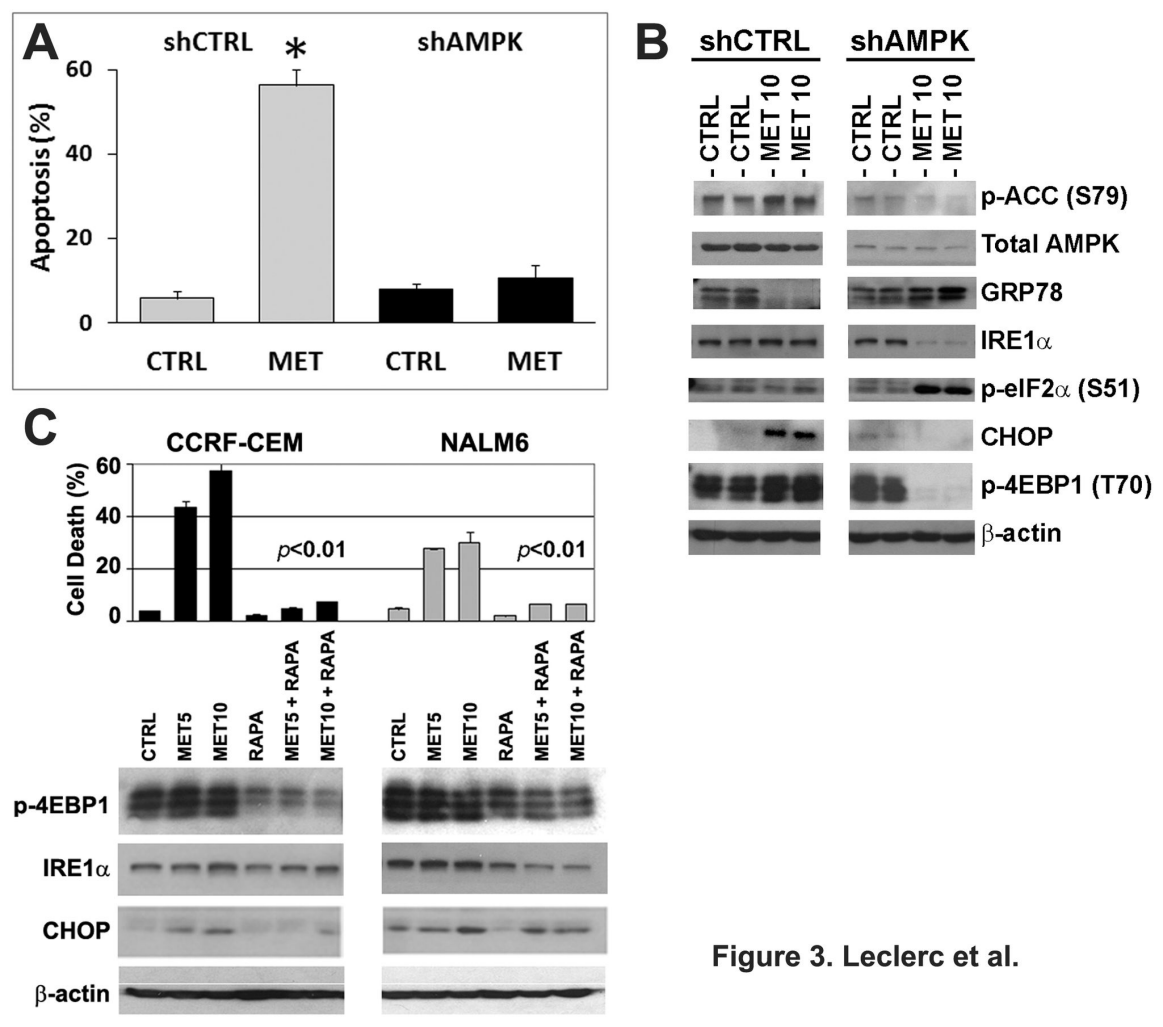
Inhibition of mTOR-dependent protein synthesis reverses metformin-induced cell death. **A**) Apoptosis in CCRF-CEM cells expressing either scramble shRNA (shCTRL) or shRNA against AMPKα1 (shAMPK) treated with metformin (MET, 10 mM) for 48 h. **B**) Immunoblotting of AMPK, Akt/mTOR, and ER stress/UPR signaling pathway proteins in the cells described in (**A**). **C**) Cell death (**upper panel**) in CCRF-CEM and NALM6 cells treated with metformin (MET, 5 and 10 mM) and rapamycin (RAPA; 0.1 µg/mL), either alone or in combination for 48 h. A statistical value of *p* < 0.01 was obtained for MET + RAPA *vs*. either control or each agent alone. The cell death was expressed as a percentage (%) of cells in the population (mean ±SEM, n = 3). Western blot analysis (**lower panel**) of p-4EBP1 (T70), IRE1α, and CHOP expression in the CCRF-CEM and NALM6 cells treated with metformin plus rapamycin described in the **upper panel**.

The described role of AMPK as suppressor of the UPR [16,19] coupled with the observed activation of AMPK in cells treated with metformin, led us to investigate AMPK’s role in the mechanism of ALL cell death induced by metformin. For this purpose, stable CCRF-CEM cells expressing either shRNA targeting AMPKα1 (shAMPK) or control shRNA (shCTRL) were treated with metformin and assayed for apoptosis at 48 h. AMPK knockdown by shRNA rescued CCRF-CEM cells from metformin-induced cell death as compared to control CCRF-CEM/shCTRL cells (Figure 3A; CCRF-CEM/shAMPK (10.7%) *vs.* CCRF-CEM/shCTRL (56.4%) cell death, *p*<0.001). As expected, shAMPK expressing cells expressed significantly lower total AMPK compared to shCTRL cells, and exhibited down-regulation of p-ACC (S79) expression confirming a functional down-regulation of AMPK signaling (Figure 3B). Signaling changes in shCTRL cells mimicked those found in wild type cell lines and primary ALL cells treated with metformin (Figure 2). Down-regulation of AMPK in metformin treated cells resulted in increased expression of GRP78 indicating ALL cells regain the ability to engage the UPR to buffer metformin’s cytotoxicity. More important, the expression of IRE1α and CHOP, two UPR markers associated with apoptosis [27], were down-regulated in shAMPK expressing cells, which correlated with the rescue phenotype (Figure 3B). These data confirm our previous data describing for the first time the role for AMPK as a negative regulator of the UPR in ALL cells [15,19]. Further Western blots revealed that AMPK knockdown led to phosphorylation/inhibition of eIF2α (S51) and dephosphorylation/activation of the negative regulator of CAP-dependent protein translation 4EBP1 (T70), suggesting that a concomitant decrease in protein synthesis also contributed to decreased metformin-induced cell death in AMPK knockdown cells (Figure 3B). Taken together, our data indicate that AMPK knockdown rescues ALL cells from metformin-induced cell death by restoring the UPR/GRP78 function, down-regulating UPR-mediated apoptotic factors, and interrupting protein synthesis.

To further assess whether the rate of protein synthesis is also an important determinant of metformin-induced cell death, we used a pharmacological approach to inhibit mTOR-dependent protein synthesis with rapamycin [42]. As shown in Figure 3C, inhibition of mTOR with rapamycin significantly decreased the sensitivity of ALL cells to metformin and, these effects correlated with decreased phosphorylation (activation) of the negative regulator of protein synthesis 4EBP1 (T70), suggesting a decrease in protein synthesis (Figure 3C). In addition, these effects in rapamycin-treated ALL cells were associated with down-regulation of the UPR apoptotic markers IRE1α and CHOP. Consequently, the AMPK knockdown data plus these findings in rapamycin-treated ALL cells support that the mechanism of metformin-induced cell death is dependent on AMPK activation, and that AMPK’s suppression of the UPR plus failure to down-regulate protein synthesis in metformin treated ALL cells undergoing proteotoxic/ER stress leads to apoptotic cell death.

### Metformin induces expression of PIM-2 and Akt as a compensatory survival mechanism in ALL

**Figure 4:**
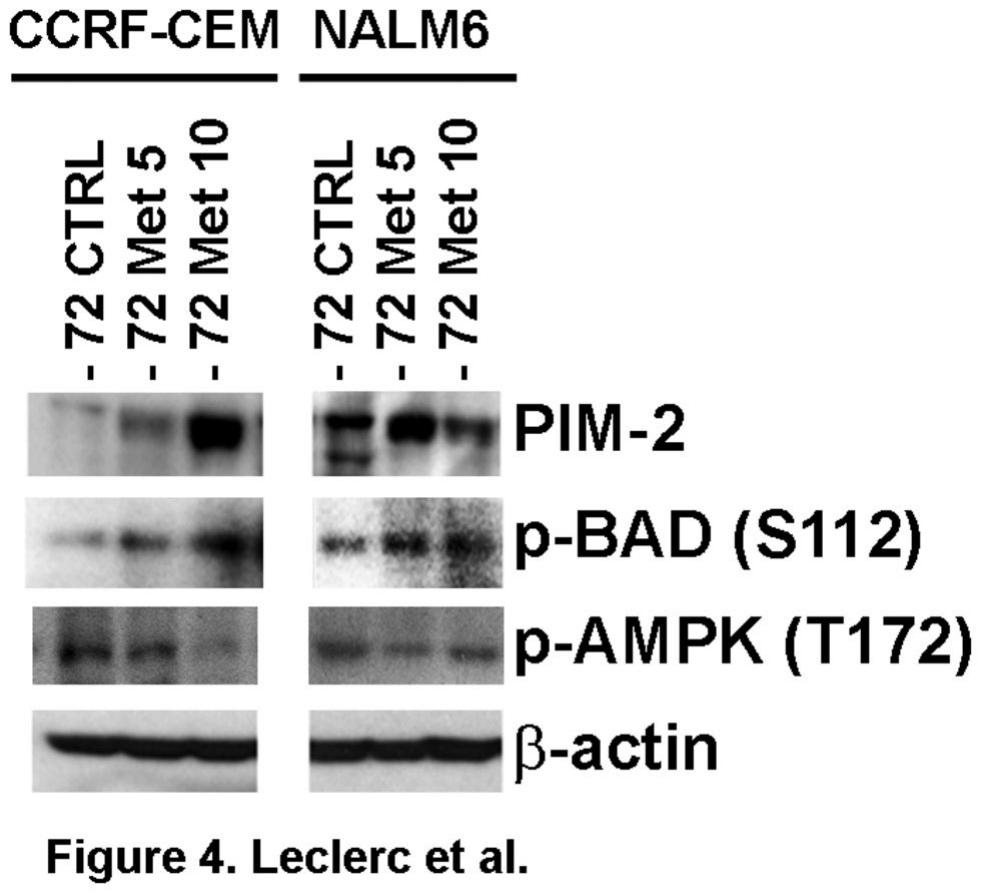
Metformin induces expression of PIM-2 in ALL cells. Western blot analysis of PIM-2, p-BAD (S112) and p-AMPK (T172) expression in CCRF-CEM and NALM6 cells treated with metformin (MET, 5 and 10 mM) for 72 h at 37°C.

**Figure 5:**
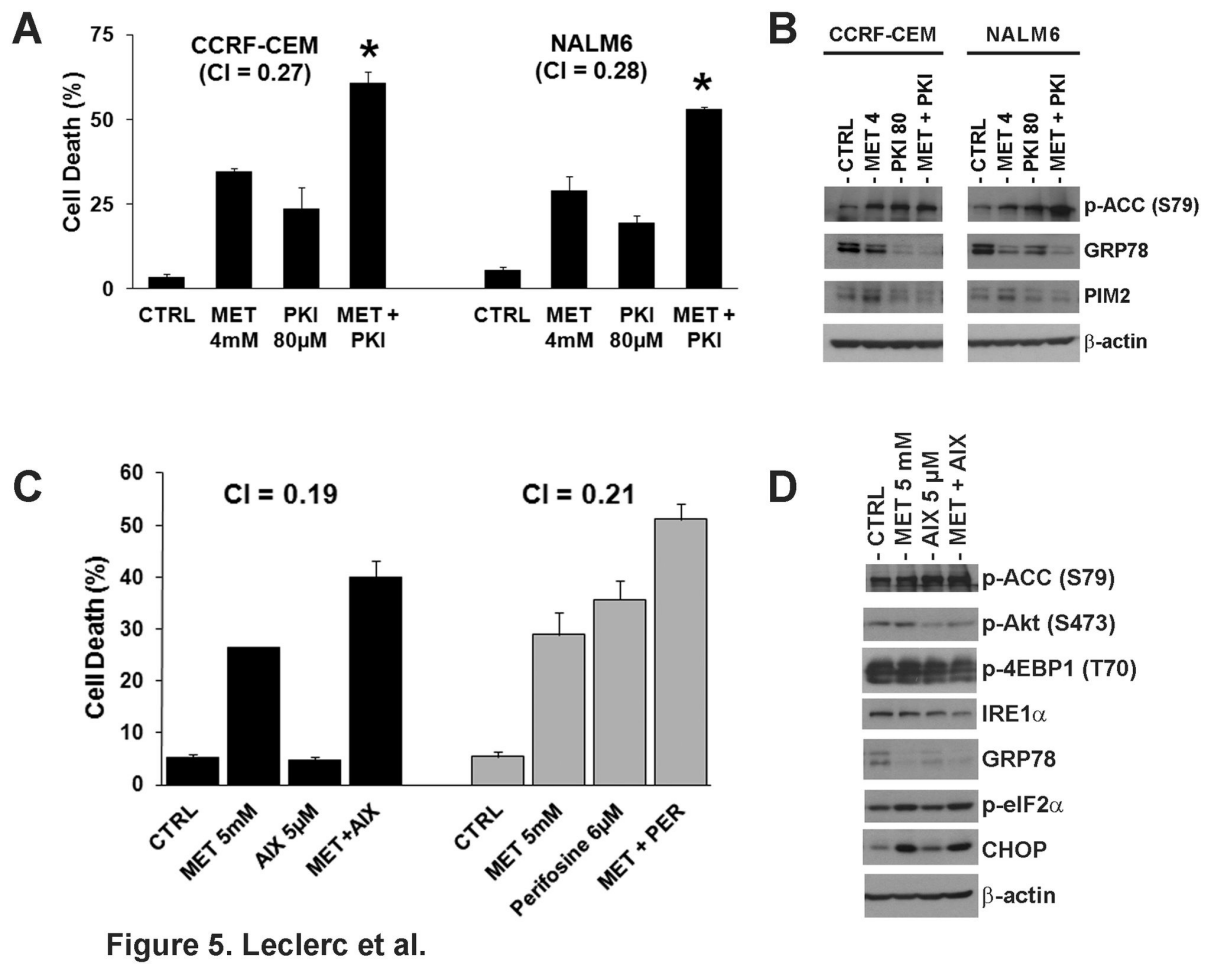
Inhibition of PIM-2 and Akt kinases synergistically sensitizes ALL cells to metformin. **A**) Cell death in CCRF-CEM and NALM6 cells treated with metformin (MET, 4 mM) and the PIM-1/2 kinase inhibitor V (PKI; 80 µM), either alone or in combination for 72 h at 37°C. The CI values of 0.27 and 0.28 indicate synergism. **B**) Immunoblotting of p-ACC (S79), GRP78, and PIM-2 expression in the CCRF-CEM and NALM6 cells treated with MET plus PKI described in (**A**). **C**) Cell death in NALM6 cells treated with metformin (MET, 5.0 mM) and Akt inhibitor X (AIX; 5 µM) or perifosine (PER; 6 µM), either alone or in combination for 72 h at 37°C. CI values of 0.19 (MET + AIX) and 0.21 (MET + PER) indicate synergism. The cell death values were expressed as a percentage (%) of cells in the population (mean ±SEM, n = 3). **D**) Immunoblotting of AMPK/ACC, Akt/mTOR, and UPR signaling pathway proteins in the NALM6 cells treated with MET (5.0 mM) plus AIX (5 µM) described in (**C**).

It has been shown that AMPK activity is regulated by multiple kinases such as LKB1 [43], PIM kinases [31], and CAMKK2 [44]. Among these, PIM kinases have been reported to regulate both AMPK and protein synthesis [45], raising the possibility that PIM kinases may interact with AMPK and play a role in the mechanism of metformin-induced cell death. To test this hypothesis, we examined the expression of PIM-2 in metformin-treated CCRF-CEM and NALM6 cells. Figure 4 shows that the expression of PIM-2 at 72 h was increased in both ALL cell lines treated with metformin, and correlated with increased phosphorylation of BAD at Ser112, indicating inhibition of the pro-apoptotic activity of BAD to promote cell survival [46]. The fact that BAD is a known downstream target of PIM-2 [32] suggests that PIM-2 is expressed to block the apoptotic events triggered by metformin. More important, we found that level of p-AMPK (T172) inversely correlated with level of PIM-2 expression resulting in inhibition of p-AMPK activity following PIM-2 up-regulation. Our findings are consistent with the described interaction between PIM kinases and AMPK in leukemia cells [31] and we hypothesized that up-regulation of PIM kinases represents a compensatory survival mechanism that is expressed in response to metformin’s cytotoxicity in ALL cells. To test the pro-survival role of PIM kinases, we evaluated the effects of a PIM-1/2 kinase inhibitor in metformin-treated ALL cells. Figure 5A demonstrates that inhibition of PIM-1/2 kinase activity using the PIM-1/2 kinase inhibitor V (PKI) synergized with metformin in inducing cell death in CCRF-CEM and NALM6 cells with CI values of 0.27 and 0.28, respectively. More important, these effects were associated with increase expression of p-ACC, a marker for AMPK activity, and down-regulation of GRP78, which were greater for the combination of metformin plus PKI (Figure 5B). These data suggest that PIM kinase activity can modulate metformin-induced cell death through its known cross-talk with AMPK which can influence the ability of ALL cells to engage the UPR in response to metformin-induced ER and proteotoxic stress. The observed changes in GRP78 expression following co-treatment with metformin and inhibitors of PIM-1/2 further support that metformin induces cell death by ER stress/UPR mediated mechanism(s) in ALL by preventing ALL cells from engaging the UPR to effectively buffer ER/proteotoxic stress.

We previously demonstrated that whereas AMPK negatively regulates the UPR, Akt up-regulates it allowing cells to better cope with ER and proteotoxic stressors [15,19]. On this basis, we used the Akt inhibitors perifosine and Akt inhibitor X (AIX), and determined their effects on metformin-induced apoptosis. NALM6 cells were treated with metformin, perifosine, AIX, or the combination of either Akt inhibitor plus metformin. Figure 5C shows that inhibition of Akt with both Akt inhibitors sensitized NALM6 cells to metformin-induced cell death. Indeed, these agents led to 40% (metformin + AIX) and 52% (metformin + perifosine) greater apoptotic death as compared to each agent alone (AIX, 4.8%; perifosine, 35.7% cell death, *p*<0.001), and these interactions were synergistic (CI=0.19 for metformin + AIX; CI=0.21 for metformin + perifosine). Immunoblots of NALM6 cells treated with AIX plus metformin showed significant down-regulation of p-Akt (S473) and increased in functional AMPK signaling (up-regulation of p-ACC (S79)) leading to down-regulation of the UPR markers GRP78 and IRE1α (Figure 5D). The magnitude of these changes was greater for the combination. These changes also correlated with greater increase in expression of p-eIF2α (S51) and CHOP, again supporting the premise that metformin’s induction of cell death is through a UPR-mediated mechanism.

## Discussion

Metformin, one of the most widely prescribed anti-diabetic drugs, has recently received significant attention as an antineoplastic agent due to its cytotoxic effects in a variety of solid tumor cancer cell lines including prostate, colorectal, lung, pancreatic, and breast [12]. Its anti-cancer effect is supported by epidemiological studies that show a decrease in cancer incidence in metformin-treated patients [14]. Although its mechanism of action in hepatic glucose metabolism is well documented, little is known about metformin’s mechanism of action in cancer cells, particularly in leukemia. It has also been proposed that most of metformin’s cytotoxic effects are mediated through activation of the AMPK pathway, the main regulator of cellular energy homeostasis [3]. Here, we show for the first time that metformin induced cell growth arrest and apoptosis in ALL cell models and primary cells occurs through induction of the ER stress/UPR-mediated cell death pathway, and that this effect is AMPK-dependent. In this context through AMPK’s effect as a negative regulator of the UPR, metformin prevents ALL cells from effectively engaging the UPR to overcome ER and proteotoxic stress-induced irreversible cellular damage leading to apoptotic death.

The notion that metformin is capable of inhibiting the UPR was first reported by Saito et al. [39], who found that antidiabetic biguanides could inhibit GRP78 activity during glucose deprivation. Our data not only confirm that metformin down-regulates GRP78 but also demonstrate that metformin induces stress in the ER lumen evidenced by activation of ATF6, IRE1α, and CHOP in metformin-treated ALL cells. It has been reported that metformin reduces the ATP/AMP ratio by targeting complex 1 of the respiratory chain [11,47]. Therefore, metformin-induced ER stress is likely triggered by accumulation of unfolded/misfolded proteins in the ER as a consequence of ATP depletion in a manner akin to glucose deprivation [48]. In various normal cell types including cardiomyocytes [17] and bovine aortic endothelial cells [18], the activation of AMPK by AICAR or metformin were found to be tissue protective via AMPK-dependent suppression of the UPR. Others have reported that the tissue protective effects of metformin in renal tubular epithelial cells were AMPK-independent [38]. In contrast, we previously demonstrated that AMPK activation by AICAR, methotrexate, or 2-DG led to inhibition of the UPR and cytotoxicity in ALL cells [15,19]. We now report that metformin’s induction of apoptosis in ALL cells is AMPK-dependent and occurs via a UPR-mediated mechanism. Therefore, tissue specificity appears to exist in the mechanism by which certain AMPK activators suppress the UPR and in addition, whereas AMPK suppression of the UPR may be beneficial to normal tissues, it induces cell death in several cancer phenotypes.

Herein, we hypothesized that concomitant persistence of metformin-induced ER stress via ATP depletion and inhibition of GRP78 following treatment with metformin leads to ALL cell death demonstrated by the increased expression of the UPR-mediated apoptotic factors IRE1α, and CHOP [25]. It has been shown that down-regulation of GRP78 expression is sufficient to induce apoptosis and plays a critical role in physiologic and pathologic stress coping mechanisms used by a variety of cell types [24]. This report is the first to elucidate the role of UPR in metformin-induced cell death in ALL and in that context it identifies AMPK as an essential regulator of this mechanism. Indeed, down-regulation of AMPK using shRNA completely abrogated metformin-induced apoptosis in ALL, and more important correlated with increased GRP78 expression and down-regulation of the UPR-mediated apoptotic factors IRE1α and CHOP. Mechanistically, this report provides further evidence and confirms our previous findings showing that AMPK acts as a physiological suppressor of the UPR whereas Akt up-regulates the UPR in ALL cells [15,19]. Therefore, our recent body of work demonstrates that in ALL cells undergoing metabolic and energetic stress, the UPR is regulated via crosstalk between AMPK and Akt, and these interactions determine the fate (i.e. death *vs.* survival) of ALL cells under conditions of metabolic and/or proteotoxic stress.

In our ALL cell models, the mechanisms responsible for the regulation of protein synthesis also appear altered following exposure to metformin. Our data showed that down-regulation of AMPK led to increased phosphorylation of p-eIF2α and dephosphorylation of p-4EBP1, both events being associated with inhibition of protein synthesis. Therefore, shRNA knockdown of AMPK rescued ALL cells through inhibition of protein synthesis coupled with up-regulation of the UPR (increased GRP78 expression). In support for this mechanism is the rescue we observed following co-treatment with the mTOR inhibitor rapamycin, a known inhibitor of CAP-dependent protein synthesis, which resulted in decreased IRE1α and CHOP expression. The relationship between protein translation and UPR activation was recently highlighted by Matsuo et al. [49], which showed that hyperactivation of 4EBP1 prevented UPR activation. Our findings do contrast with the recent report by Grimaldi et al. [50] proposing that metformin induces cell death by blocking the catalytic activity of mTORC1 and repressing mRNA translation, although these investigators did not assess the role of the UPR in their model. Based on our data, we propose that it is the balance between proteotoxic stress and UPR activity that results in either death or survival from metformin’s cytotoxicity in ALL cells.

**Figure 6:**
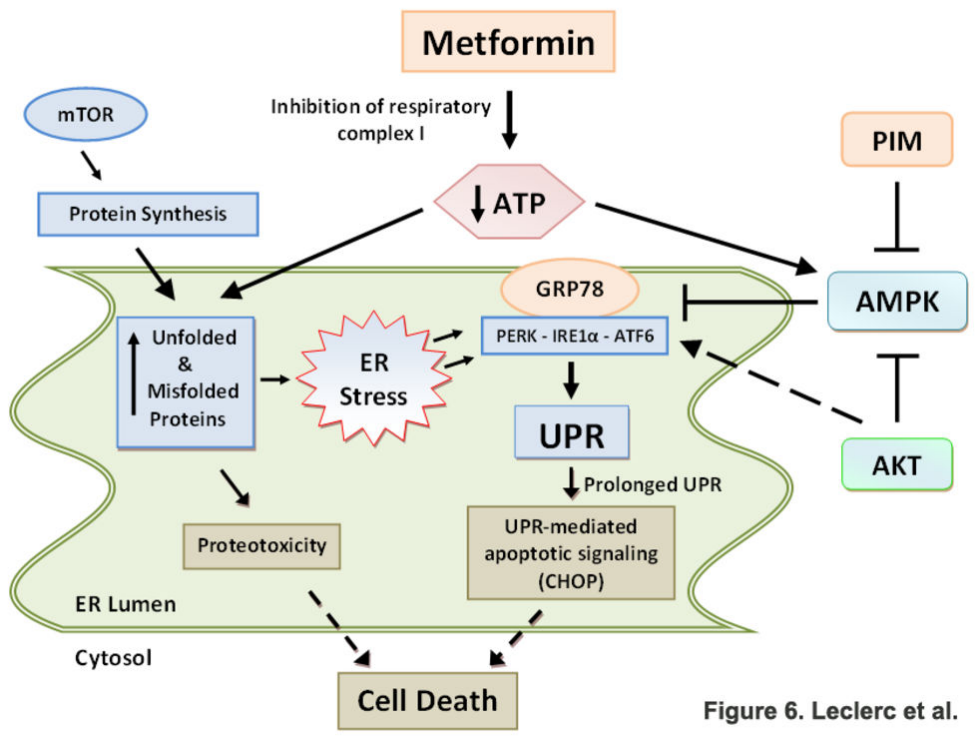
Proposed mechanism of action for metformin in ALL cells. Metformin induces metabolic stress by decreasing the ATP: AMP ratio, which leads to activation of AMPK, and increased level of unfolded/misfolded proteins in the ER lumen. The inability of ALL cells to engage the UPR caused by AMPK-dependent down-regulation of GRP78 leads to ER stress/UPR mediated cell death. The survival PIM and Akt kinases are expressed as compensatory survival mechanisms in response to metformin’s cytotoxicity to down-regulate AMPK allowing cells to effectively engage UPR and process the ER stress.

To our knowledge, the induction of PIM kinase activity by metformin had not been reported. Herein, we interpreted that metformin induced PIM-2 kinase expression is a compensatory survival mechanism due to its anti-apoptotic role via down-regulation of BAD [32]. Further, it has been demonstrated that PIM kinases can negatively regulate AMPK activity [31] and promote hematopoietic cell growth and survival [45]. The synergistic effects observed between metformin and PIM kinase inhibition support our postulate that the increase in PIM-2 expression represents a buffering response to metformin. More important, this combination led to synergistic induction of cell death. Based on our findings, we propose a new role for PIM-2 kinase as capable of modulating UPR via down-regulation of AMPK activity. We also found that targeting Akt, another survival kinase capable of modulating AMPK activity through phosphorylation of Ser485 [51], resulted in significant synergistic cell death when combined with metformin. Our data confirm our report demonstrating antagonistic roles for Akt and AMPK in modulating the UPR [19]. Based on data presented herein, we propose a model for metformin-induced cell death in ALL cells via ER stress/UPR-mediated apoptosis in which metformin, by decreasing the ATP/AMP ratio promotes i) ATP depletion and unfolded protein accumulation in the ER lumen resulting in proteotoxic and ER stress, and ii) AMPK activation which leads to suppression of the UPR (down-regulation of GRP78), leading to UPR-mediated apoptotic cell death (Figure 6). In support of this proposed model we present evidence that down-regulation of AMPK rescues ALL cells from metformin-induced apoptosis by interrupting protein synthesis and increasing UPR activity, which would reduce the load of unfolded proteins in the ER and restore the ability of the cells to cope with the ER/proteotoxic stress.

In summary, our studies demonstrate that the crosstalk between AMPK, Akt, PIM-2 kinase, and UPR signaling pathways determines metformin-induced cell death in ALL. We demonstrate for the first time that metformin induced apoptosis in ALL lymphoblasts occurs via UPR-mediated mechanisms which are entirely AMPK-dependent. We also provide further evidence for the roles of AMPK and Akt as regulators of the UPR in ALL. Finally, our data not only demonstrate the ability of metformin to induce significant cell death in ALL cell lines and primary cells supporting future translation into clinical trials, but also uncover strategies exploiting synthetic lethality by combining metformin and selective inhibitors of these pathways that may also be suitable for clinical translation in patients with ALL.
